# A combined 4D flow MR imaging and fluid–structure interaction analysis of ascending thoracic aortic aneurysms

**DOI:** 10.1007/s10237-025-01939-6

**Published:** 2025-03-11

**Authors:** Yu Zhu, Chlöe Armour, Binghuan Li, Selene Pirola, Yousuf Salmasi, Thanos Athanasiou, Declan P. O’Regan, Xiao Yun Xu

**Affiliations:** 1 Department of Chemical Engineering, Imperial College London, London, UK; 2 National Heart and Lung Institute, Imperial College London, London, UK; 3 Department of Biomechanical Engineering, Delft University of Technology, Delft, Netherlands; 4 Department of Surgery and Cancer, Imperial College London, London, UK; 5 MRC London Institute of Medical Sciences, Imperial College London, London, UK

**Keywords:** Fluid–structure interaction, Ascending thoracic aortic aneurysm, Four-dimensional flow magnetic resonance imaging, Inlet velocity profiles

## Abstract

This study aimed to characterize the altered hemodynamics and wall mechanics in ascending thoracic aortic aneurysms (ATAA) by employing fully coupled two-way fluid–structure interaction (FSI) analyses. Our FSI models incorporated hyperelastic wall mechanical properties, prestress, and patient-specific inlet velocity profiles (IVP) extracted from 4D flow magnetic resonance imaging (MRI). By performing FSI analyses on 7 patient-specific ATAA models and 6 healthy aortas, the primary objective of the study was to compare hemodynamic and biomechanical features in ATAA versus healthy controls. A secondary objective was to examine the need for 4D flow MRI-derived IVP in FSI simulations by comparing results with those using two commonly adopted idealized IVPs: Flat-IVP and Para-IVP for selected cases. Our results show that, compared to the healthy aortas, the ATAA models exhibited highly disturbed blood flow in the ascending aorta. Consequently, maximum turbulent kinetic energy (TKE) at peak systole (155.0 ± 188.4 Pa) and maximum time-averaged wall shear stress (TAWSS) (8.6 ± 6.5 Pa) were significantly higher in the ATAA cohort, compared to 0.6 ± 0.5 Pa and 2.8 ± 0.7 Pa in the healthy aortas. Peak wall stress was also nearly doubled in the ATAA group (414 ± 108 kPa vs. 215 ± 31 kPa). Additionally, comparisons of simulation results across models with different IVPs underscore the importance of prescribing 3D-IVP at the inlet, especially for ATAA cases. Using idealized IVPs in two selected ATAA models (P1 and P7) substantially reduced the maximum TKE from 571 Pa to 0.01 Pa (Flat-IVP) and 0.02 Pa (Para-IVP) in P1 and from 73 Pa to 0.01 Pa (Flat-IVP) and 0.08 Pa (Para-IVP) in P7, while the maximum TAWSS in the ascending aorta decreased from 9.6 Pa to 0.7 Pa (Flat-IVP) and 0.9 Pa (Para-IVP) in P1, and from 3.6 Pa to 1.2 Pa and 0.9 Pa, respectively, in P7. Moreover, idealized IVPs also caused the peak wall stress to reduce by up to 11.5% in P1 with severe aortic valve stenosis, and by up to 2% in P7 with mild aortic regurgitation. These results highlight the importance of FSI simulations combined with 4D flow MRI in capturing realistic hemodynamic and biomechanical changes in aneurysmal aortas.

**Supplementary Information:**

The online version contains supplementary material available at 10.1007/s10237-025-01939-6.

## Introduction

Ascending thoracic aortic aneurysm (ATAA) is a degenerative disease characterized by the permanent dilatation of the ascending aortic wall. ATAAs are silent but lethal since they are seldomly detected before an acute event, such as a dissected or a ruptured aneurysm. A dissection originating in the ascending aorta has been reported to cause a mortality rate of 15–30% (Trimarchi et al. 2005), which can increase to over 94% if an aortic rupture occurs (Martufi et al. 2014). Once an ATAA is confirmed, clinicians typically rely on the maximum diameter to assess the risk of rupture. Surgical repair is generally recommended for asymptomatic aneurysms when the maximum diameter reaches 5.5 cm or greater (Isselbacher et al. 2022). However, nearly 60% of ascending aortic dissections have been reported to occur at diameters less than 5.5 cm (Pape et al. 2007).

Evaluations of patient-specific wall stress using finite element analysis (FEA) have been reported to assist in risk stratification for ATAA patients by incorporating either patient-specific (García-Herrera et al.^2017^; Trabelsi et al. 2015; Wisneski et al. 2014) or literature-based (Xuan et al. 2018; Wang et al. 2021) wall material properties. Abnormal hemodynamic conditions predicted by computational fluid dynamics (CFD) have also been correlated with ATAA progression (Gülan et al. 2018; Jayendiran et al. 2020; Ramaekers et al. 2024). In particular, wall shear stress (WSS) coupled with mechanobiological processes offers valuable insights into the progression of ATAAs, potentially aiding in predicting aneurysm growth and rupture risk (Condemi et al. 2017, 2019; McClarty et al. 2022; Mousavi et al. 2021; Salmasi et al. 2021).

The aforementioned FEA studies typically impose a uniform blood pressure as the loading condition, whereas CFD studies assume a rigid aortic wall. Fluid–structure interaction (FSI) approach is preferable as it accounts for dynamic interactions between blood flow and the aortic wall. FSI simulations have been reported to outperform CFD in distinguishing between Marfan syndrome patients with stable and unstable aortic dilatations (Pons et al. 2020). Comparative FSI studies between ATAAs and healthy aortas suggest that aneurysmal dilatations significantly alter hemodynamics, potentially increasing the risk of rupture due to elevated wall stress and deformation (Petuchova and Maknickas 2022; Taheri et al. 2022). Campobasso et al. (2018) assessed the impacts of aortic stiffness and peripheral resistance on wall stress predictions, suggesting that ATAA patients with stiffer walls are at a higher risk of aneurysm rupture, particularly under hypertensive conditions. However, these FSI models either employed idealized inlet velocity profiles (Petuchova and Maknickas 2022; Taheri et al. 2022) or treated the aortic wall as a linear elastic material (Campobasso et al. 2018; Petuchova and Maknickas 2022), limiting their accuracy in capturing realistic hemodynamic and biomechanical behavior. This study aims to establish fully coupled two-way FSI simulations incorporating hyperelastic material properties for the aortic wall, prestressing, and patient-specific 3D inlet velocity profiles (3D-IVP) extracted from four-dimensional flow magnetic resonance imaging (4D flow MRI). By integrating all these key elements, the altered hemodynamics and wall mechanics in ATAAs can be faithfully characterized, allowing more reliable comparisons with healthy controls.

In addition, the use of idealized inlet velocity profiles has been shown to significantly influence aortic hemodynamics in both healthy (Morbiducci et al. 2013) and diseased aortas (Armour et al. 2021; Pirola et al. 2018; Youssefi et al. 2018). These studies highlighted the importance of using patient-specific 3D velocity profiles as inlet boundary conditions, especially for patients with abnormal aortic valves (Pirola et al. 2018; Youssefi et al. 2018). However, to the best of our knowledge, the impact of employing idealized velocity profiles on predicted hemodynamic and biomechanical indices has not been comprehensively quantified in a FSI framework for ATAA models.

By performing FSI analyses on 7 patient-specific ATAA models and 6 healthy aortas, the primary objective of this study is to compare hemodynamics and biomechanics between ATAA models and healthy aortas. The secondary objective is to assess the effect of using idealized inlet velocity profiles, namely flat inlet velocity profiles (Flat-IVP) and parabolic velocity profiles (Para-IVP), on the predicted hemodynamic and biomechanical quantities. This was achieved by performing additional FSI simulations on three selected models, one from the group of healthy aortas and two from the ATAA cohort.

## Material and methods

### Data acquisition

The study was ethically approved (17/NI/0160) by the Health Research Authority (HRA) in the UK. MRI scans were performed on 7 ATAA patients and 6 healthy volunteers using a 3T MRI scanner (Siemens Healthcare, Erlangen, Germany). High-resolution computed tomography angiography (CTA) images of the ATAA were also available for geometric reconstruction, with a slice thickness and increment of 0.625 mm. Geometric models of the healthy aortas were reconstructed from 4D flow MR images with a spatial resolution in the following range: (1.9–2) mm × (1.88–1.98) mm × (1.88–1.98) mm. All 4D flow MRI scans recorded either 20 or 25 time points within a cardiac cycle. Key characteristics of the ATAA patients are summarized in Table 1.

**Table 1:** Characteristics for patients with ATAA

	AV morphology	AV hemodynamic	Brachial pressures (systolic/diastolic)	Surgery
1	Tri-leaflet	Severe AS	140/108	AVR + ascending replacement
2	Tri-leaflet	Severe AR	139/86	Bentall procedure
3	Tri-leaflet	Severe AR	153/60	AVR + ascending replacement + FET
4	Tri-leaflet	Moderate AR	120/76	AVR + ascending replacement
5	Tri-leaflet	Severe AR	144/92	AVR + ascending replacement
6	Tri-leaflet	Moderate AR	131/60	AVR + ascending replacement
7	Tri-leaflet	Mild AR	124/88	Bentall procedure

AR, aortic regurgitation; AS, aortic stenosis; AV, aortic valve; AVR, aortic valve replacement; FET, frozen elephant trunk

### Geometry reconstruction and mesh generation

**Fig. 1:**
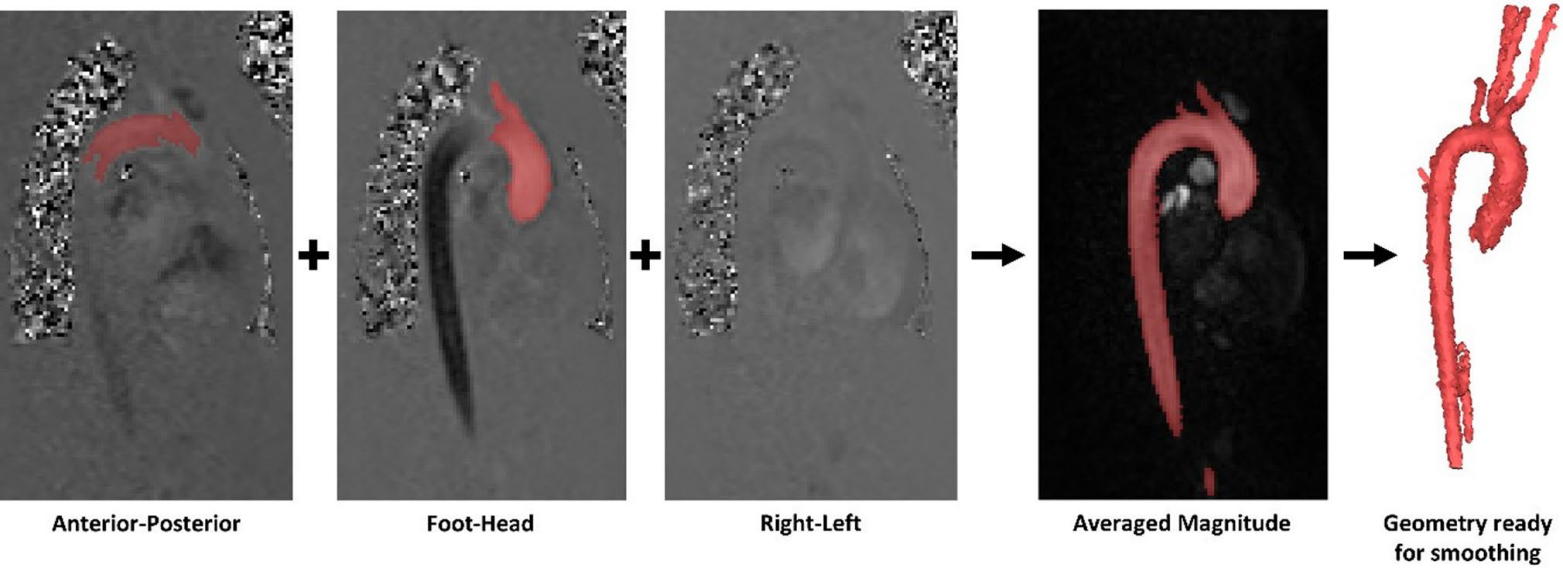
Reconstruction of healthy aorta from 4D flow MRI data. Segmentations from each velocity phase are combined on the magnitude image to form a complete 3D model

**Fig. 2:**
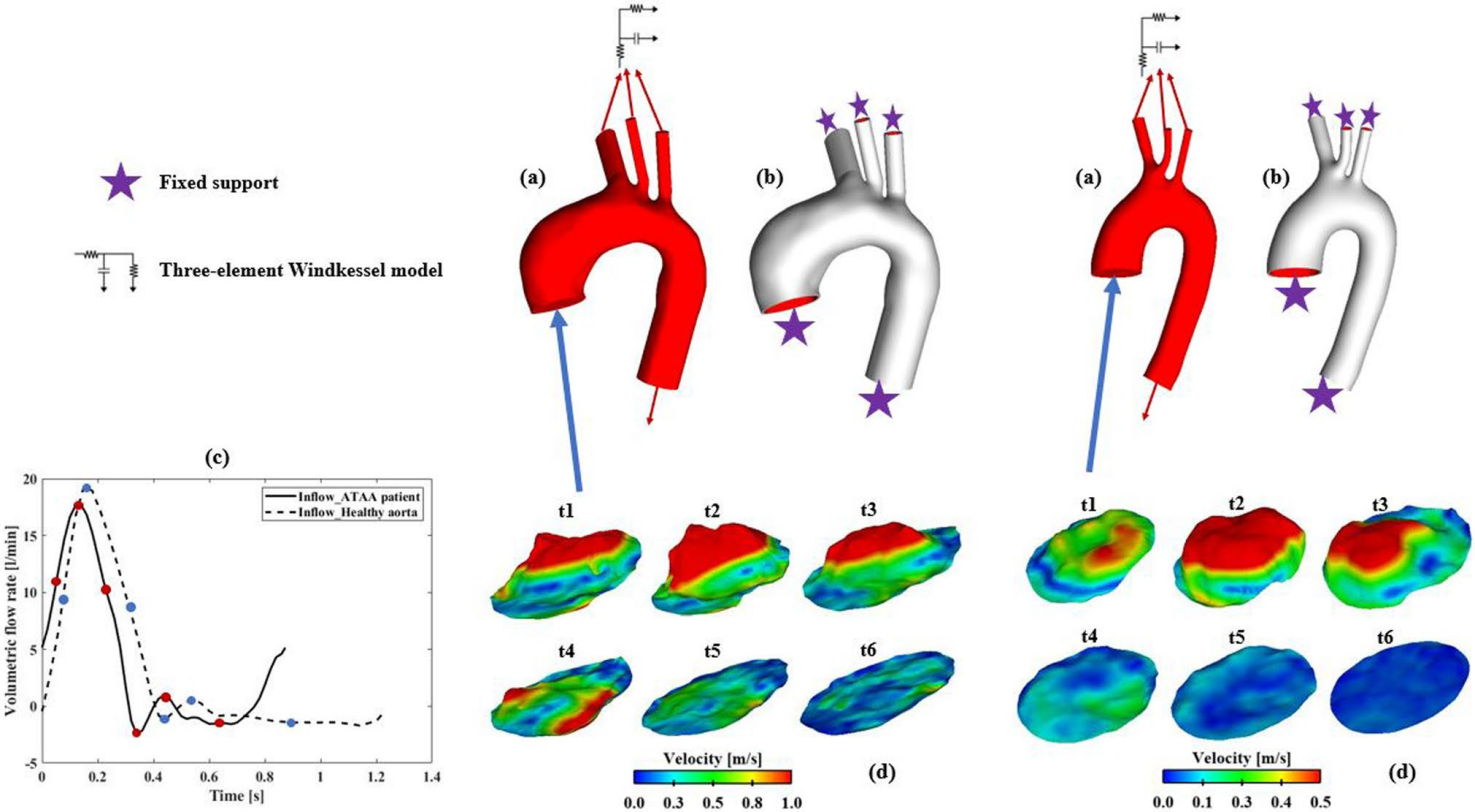
Reconstructed computational models for an ATAA patient (top left) and a healthy aorta (top right), with (**a**) the fluid domains shown in red and (**b**) the solid domains shown in white. A 3-element Windkessel model was applied at all outlets (red arrows) of the fluid domain, whereas zero-displacement constraints (purple stars) were applied at inlet and outlets of the solid domain. (**c**) Volumetric flow rate waveforms derived from 4D flow MRI of the ATAA patient and healthy aorta. (**d**) 3D inlet velocity profiles for both models at 6 representative time points (t1–t6) across the cardiac cycle. Time points t1–t6, spanning from mid-systolic acceleration to end diastole, are indicated by red and blue circles for ATAA patient and healthy aorta, respectively, on flow rate waveforms in (**c**)

Geometric models of 7 ATAA and 6 healthy aortas were reconstructed using Mimics 24.0 (Materialise, Leuven, Belgium). For both the CT and MR images, in-built automated segmentation functions were used to isolate the ascending aorta including mask thresholding and region splitting. Manual refinement was then performed to remove any small vessels such as the coronary arteries that were picked up. Specifically for geometric reconstruction of the healthy aorta, the aorta was segmented on all 3 velocity phase images and a single magnitude image, with the resulting 4 segmentations combined into one complete aortic geometry. Figure 1 demonstrates this process, with clear regions identified on the foot–head and anterior–posterior directions. Due to the uniformity of the healthy aortas, little information was captured in the right–left direction. Each segmentation from all velocity phases was then combined on the magnitude image to ensure a complete aorta was reconstructed. The reconstructed geometries were smoothed in Mimics based on a cubic spline algorithm. As shown in Fig. 2a, the reconstructed geometry served as both the 3D fluid domain and the inner surface of the wall in the FSI model. The wall structural domain was created by uniformly offsetting the inner wall surface by 2.1 mm for the ATAA models (Zhu et al. 2024) and 1.5 mm for the healthy aortas (Mensel et al. 2014).

Ansys ICEM 19.2 (ANSYS, Canonsburg, PA, United States) was used to generate the mesh. For each model, the fluid domain was meshed with a tetrahedral core and 10 prismatic layers at the wall, whereas the solid domain was discretized into unstructured tetrahedral elements. The grid convergence index (GCI) was calculated to ensure the solution was mesh independent. Further details on the mesh sensitivity analysis can be found in Supplementary material S1. The final meshes comprised approximately 2.3–3.6 million elements for the fluid domains of the ATAA models and 0.8–1.1 million elements for the healthy aortas. Regarding the solid domain, the final meshes contained 1.2–1.8 and 0.7–0.8 million elements for the ATAA and the healthy aortas, respectively.

### Fluid domain

Blood was assumed to be incompressible with a constant density of 1060 kg/m^3^, and its non-Newtonian behavior was described using the empirical Carreau–Yasuda model:

1 $$\mu \left( {\dot{\gamma }} \right) = \mu_{\infty } + \left( {\mu_{0} - \mu_{\infty } } \right)\left( {1 + \left( {\lambda \dot{\gamma }} \right)^{a} } \right)^{{\frac{n - 1}{a}}}$$

where $\mu_{\infty }$ and $\mu_{0}$ are the infinite shear viscosity and the zero shear viscosity with values being 0.0035 Pa s and 0.1600 Pa s, respectively, $\dot{\gamma }$ is the shear rate, and $a$, $n$, and $\lambda$ are empirical constants with values being 0.64, 0.2128, and 8.2 s, respectively (Abraham et al. 2005). To account for possible turbulence effect, the hybrid $k - \varepsilon /k - \omega$ shear stress transport transitional model (Menter et al. 2006) was applied.

For each computational model (ATAA and healthy aorta), patient-specific 3D-IVP was extracted from 4D flow MRI data following our previously published methodology (Saitta et al. 2023; Armour et al. 2021; Pirola et al. 2018). This method involved processing the raw DICOM data using in-house Python codes to write out complete 3D volumes of velocity data at every time point from the 4D flow MRI scan. The velocity data that lie on the plane of the model inlet mesh were then extracted. These velocity data were interpolated in time to increase the temporal resolution to that of the simulation (0.001 s). The resulting 3D-IVPs were then imposed as inlet boundary conditions. Figure 2d shows examples of 3D-IVPs for an ATAA and healthy model at 6 representative time points.

Three-element Windkessel models (3-EWM) were applied at each outlet. Since extracting flow rate for each arch vessel directly from 4D flow MRI was challenging due to its limited spatial resolution, the total blood flow to the arch vessels was calculated as the difference in average flow rate, measured using 4D flow MRI, between two planes placed proximally and distally to the aortic arch, respectively (Gallo et al. 2012). The flow split among the three arch vessels was then determined based on their cross-sectional areas, and the estimated flow rate for each arch branch was used to tune the 3-EWM parameters. Moreover, patient-specific brachial pressures (Table 1) were converted to central blood pressures (Izzo 2014).

Additional FSI simulations were conducted for three selected models, one from the group of healthy aortas and two from the ATAA cohort, using either a Flat-IVP or a Para-IVP at the inlet, as described by Eqs. (2) and (3), respectively. It should be mentioned that the two selected ATAA cases (P1 and P7, as shown in Table 1) presented with severe aortic valve stenosis and mild aortic valve regurgitation, respectively. The healthy aorta was randomly selected as all the healthy volunteers exhibited normal aortic valve functions.

2 $$u\left( t \right) = \frac{Q\left( t \right)}{A}$$

3 $$u\left( {t,r} \right) = u_{{{\text{max}}}} \left( t \right) \cdot \left( {1 - \left( {\frac{r}{{R_{{{\text{eq}}}} }}} \right)^{2} } \right)$$

where $Q$ is the time-varying inlet volumetric flow rate derived from 4D flow MRI (Fig. 2c) and $A$ is the inlet surface area. $r = \sqrt {\left( {x - x_{c} } \right)^{2} + \left( {y - y_{c} } \right)^{2} + \left( {z - z_{c} } \right)^{2} }$ is the radial position with $\left( {x_{c} , y_{c} , z_{c} } \right)$ being the coordinates of the inlet plane centroid; $R_{{{\text{eq}}}}$ is the equivalent radius of the inlet surface; and $u_{{{\text{max}}}} = \frac{2Q}{A}$ denotes the time-varying peak velocity of the parabolic profile.

### Solid domain

The aortic walls were described by the second-order Yeoh hyperelastic material model:

4 $$W = C_{10} \left( {I_{1} - 3} \right) + C_{20} \left( {I_{1} - 3} \right)^{2}$$

where *W* is the strain energy density and $I_{1}$ is the first deviatoric invariant. The material parameters, $C_{10}$ and $C_{20}$, were set to be 98.8 kPa and 779.1 kPa for ATAA walls, and 115.4 kPa and 177.4 kPa for healthy aortic walls, based on a previous study (Vorp et al. 2003). Zero-displacement constraints were applied at the inlet and at all ends of the solid domain (Fig. 2b). Empirical Rayleigh damping ($\alpha = 50, \beta = 0.1$) was also applied to account for support provided by the surrounding tissue (Zhu et al. 2022).

Prestress was estimated for each model to account for physiological initial loading state. Ansys Static Structural (ANSYS, Canonsburg, PA, United States) was used to calculate prestress, following the method described by Votta et al. (2017) and modified by Caimi et al. (2020). Briefly, the structural domain was deformed by the diastolic pressure $(P_{{{\text{dias}}}} )$, and the corresponding Cauchy stress tensor was exported and prescribed as initial stress state for the next simulation. To prevent unrealistic deformation resulting from applying the full $P_{{{\text{dias}}}}$ directly, the pressure was gradually increased in 10 increments, with each increment defined as $\Delta P = P_{{{\text{dias}}}} /10$. The procedure was repeated for each $\Delta P$ until the maximum deformation was less than 0.5 mm. Consequently, the prestress tensor equivalent to the diastolic phase was obtained and applied in the final FSI simulations.

### Fluid–structure interaction

Two-way FSI simulations were performed using ANSYS system coupling (ANSYS, Canonsburg, PA, US), which couples ANSYS Structure (solid solver) and ANSYS CFX (fluid solver) through a partitioned approach. Rigid-wall CFD simulations were firstly performed for 6–9 cycles for each model to reach a periodic stable solution. The results from the last cycle were then used as the initial condition for FSI simulations, which allowed the FSI simulation to reach periodic stability within 3 cycles. A high-order advection scheme was adopted for spatial discretization of the conservation equations and a second-order implicit backward Euler scheme was chosen for temporal discretization, with a fixed time-step of 0.001 s. The solution convergence was controlled by setting the maximum RMS residual as 1e−5. During the FSI simulation, the updated mesh was smoothed using displacement diffusion method with mesh stiffness blended with distance and small volumes. An under-relaxation factor of 0.6 was specified to help converge the solutions. Within each coupled time-step (0.001 s), iterations were repeated until the maximum number of iterations was reached or the data transferred between solvers converged, with the maximum RMS residual lower than 0.01. Results obtained in the last cycle were obtained for detailed analysis using CEI Ensight 10 (CEI Inc, Apex, NC, US).

### Post-processing of results

Kinetic energy (KE) is used to quantify high energy regions that correlate with dominant flow features, whereas turbulent kinetic energy (TKE) indicates the level of turbulence. Following relevant studies in the literature (Lantz et al. 2013; Manchester et al. 2021), KE and TKE can be evaluated by multiplying their standard values by the blood density as follows:

5 $${\text{KE}} = \frac{\rho }{2} \mathop \sum \limits_{i} v_{i}^{2}$$

6 $$TKE = \frac{\rho }{2} \mathop \sum \limits_{i} v_{i}{\prime}^{2}$$

where $\rho$ is the blood density, $v_{i}$ is the velocity component with $i = 1, 2, 3,$ and $v_{i}{\prime}$ is the fluctuating velocity component. Using the above expressions, KE and TKE are expressed in the unit of pascal.

Time-averaged WSS (TAWSS) was calculated by averaging WSS magnitude over a cardiac cycle, as defined by Eq. (7). TAWSS represents the mean shear load over time, accounting for its cumulative effect on the vessel wall over the entire cardiac cycle.

7 $${\text{TAWSS}} = \frac{1}{T}\mathop \smallint \limits_{0}^{T} \left| {\tau_{\omega } } \right|{\text{d}}t$$

Flow asymmetry index (${\text{Flow}}_{{{\text{asymmetry}}}}$) and flow dispersion index (${\text{Flow}}_{{{\text{dispersion}}}} )$ were also computed and compared among different IVPs to quantitatively describe changes in flow morphology (Youssefi et al. 2018).

${\text{Flow}}_{{{\text{asymmetry}}}}$, defined in Eq. (8), was calculated at several cross-sectional planes along the aorta to assess the level of flow eccentricity.

8 $${\text{Flow}}_{{{\text{asymmetry}}}} = \frac{||{{\mathbf{x}}_{{{\text{plane}}}} - {\mathbf{x}}_{{v_{{{\text{max}}, 15}} }}|| }}{{R_{{{\text{eq}}}} }} \times 100\%$$

where ${\mathbf{x}}\left( {x, y, z} \right)$ denotes the coordinates of the geometric centroid, with the subscript $plane$ and $v_{{{\text{max}},15}}$ referring to the defined cross-sectional aortic plane and an area in the plane that contains the top 15% of peak systolic velocity, respectively. The Euclidian distance (||·||) between these two coordinates is then divided by the equivalent radius of the cross-sectional plane ($R_{{{\text{eq}}}}$). ${\text{Flow}}_{{{\text{asymmetry}}}}$ ranges from 0 to 100%, where 0% indicates that flow is perfectly central to the axis of the vessel and 100% indicates completely eccentric flow.

${\text{Flow}}_{{{\text{dispersion}}}}$ was calculated based on Eq. (9), representing whether the flow on each plane is peaked or broad.

9 $${\text{Flow}}_{{{\text{dispersion}}}} = \frac{{A_{{v_{{{\text{max}}, 15}} }} }}{{A_{{{\text{plane}}}} }} \times 100\%$$

where $A_{{v_{{{\text{max}}, 15}} }}$ and $A_{{{\text{plane}}}}$ correspond to the areas of the top 15% of peak systolic velocity and the cross-sectional plane, respectively. The value of ${\text{Flow}}_{{{\text{dispersion}}}}$ positively correlates with the breadth of the peak velocity distribution.

Finally, the maximum principal stress was calculated to compare the wall stress distribution.

## Results

### Comparisons between ATAA patients and healthy aortas

#### Flow patterns

**Fig. 3:**
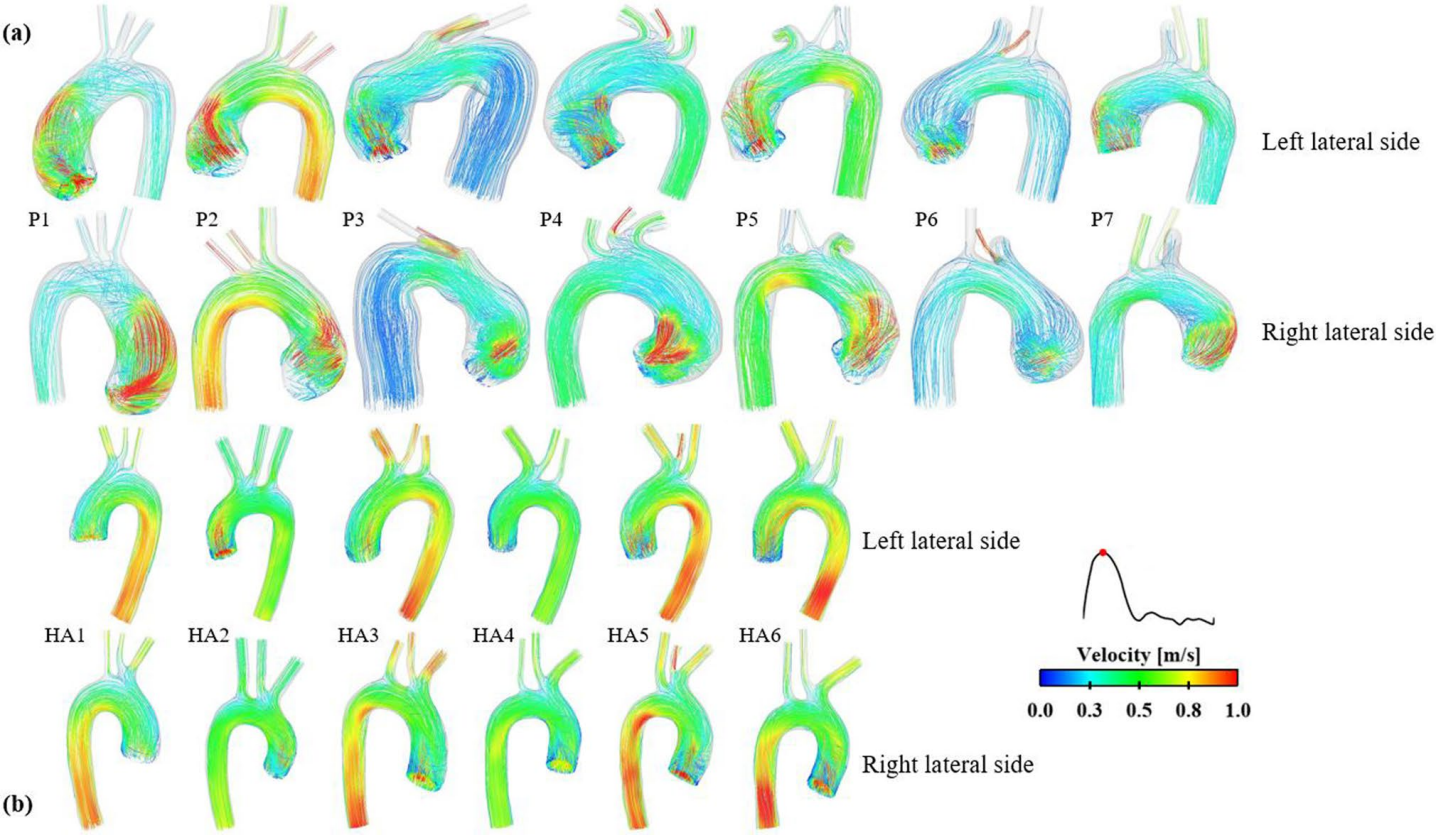
Comparison of instantaneous velocity streamlines at peak systole between (**a**) patients with ascending thoracic aortic aneurysm (ATAA) and (**b**) healthy aortas (HAs)

The predicted flow patterns visualized through instantaneous velocity streamlines at peak systole are shown in Fig. 3. In the healthy aortas (Fig. 3b), blood flow is more organized with peak velocities deviating less from the aortic centerline in the ascending aorta. The central high-velocity jet flow originating from the ascending aorta smoothly extends toward the aortic arch where flow is influenced by the arch curvature. In contrast, blood flow in the aneurysmal ascending aorta is more disturbed. A common feature across all ATAA models is that the jet flow is skewed anteriorly, impinging on the aneurysmal wall (Fig. 3a), causing highly rotational flow in the center of the aneurysm.

#### Kinetic energy

**Fig. 4:**
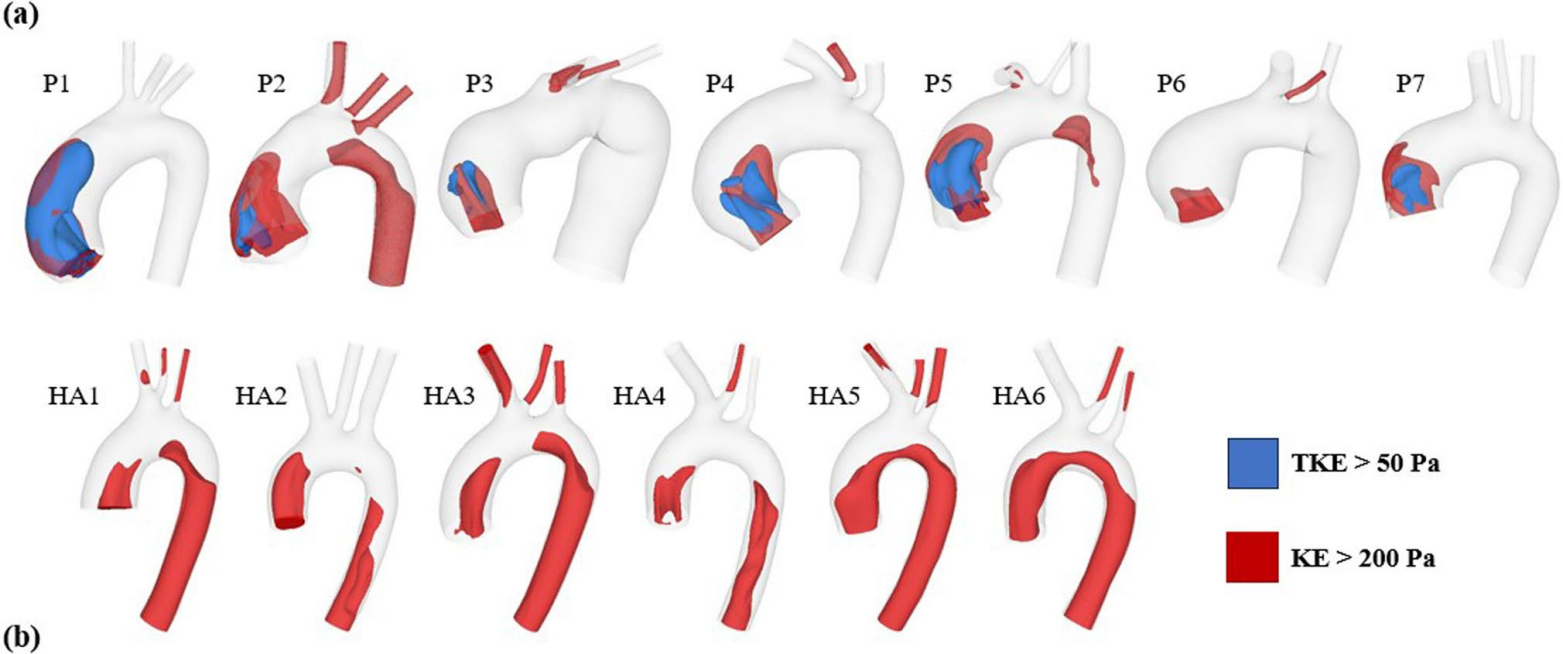
Iso-volumes of kinetic energy (KE) > 200 Pa and turbulent kinetic energy (TKE) > 50 Pa at peak systole were compared between (**a**) ATAA patients and (**b**) healthy aortas. KE > 200 Pa and TKE > 50 Pa are displayed in red and blue, respectively

The iso-volumes of KE > 200 Pa and TKE > 50 Pa at peak systole were calculated, and their comparisons between ATAA patients and healthy aortas are shown in Fig. 4. Instantaneous time point of peak systole is selected since this phase typically exhibits the most pronounced and physiological relevant turbulent characteristics, making it the most representative for assessing TKE. High levels of KE are observed in both the ascending and descending portions of the healthy aortas, whereas in most ATAA cases, they are primarily found in the ascending aorta. However, none of the healthy aortas exhibit regions with high levels of TKE. In contrast, regions with TKE > 50 Pa are observed in 6 of 7 ATAA models, generally coinciding with regions of high KE, as shown in Fig. 4a. Quantitatively, the maximum TKE ranges from 19.4 Pa to 571 Pa in the ATAA models, compared to 0.1 Pa to 1.1 Pa in the healthy aortas.

#### Time-averaged wall shear stress

**Fig. 5:**
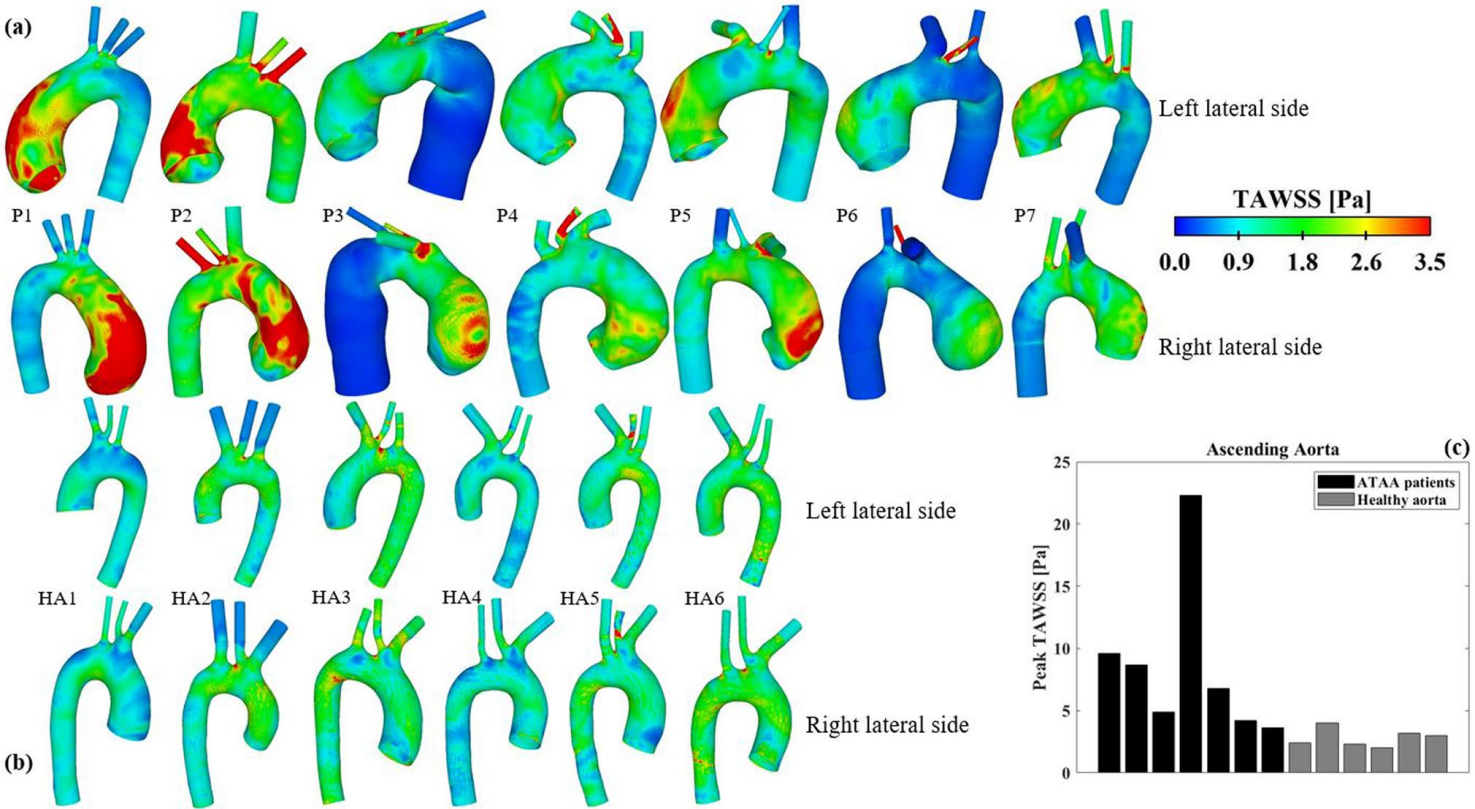
Comparison of time-averaged wall shear stress (TAWSS) distributions between (**a**) ATAA patients and (**b**) healthy aortas. (**c**) Quantitative comparison of peak TAWSS in the ascending aorta between the two groups

Figure 5 compares the TAWSS distributions between the two groups. Similar to the flow patterns, TAWSS is more uniformly distributed along the healthy aortas, while markedly high values of TAWSS are observed along the outer curvature of the aneurysmal aortas, resulting from jet flow impingement. Consequently, the peak TAWSS in the ascending aorta is significantly higher in ATAA patients (Fig. 5c), ranging from 3.6 Pa to 22.3 Pa, than the controls, which ranges from 2.0 Pa to 3.2 Pa.

#### Wall stress

**Fig. 6:**
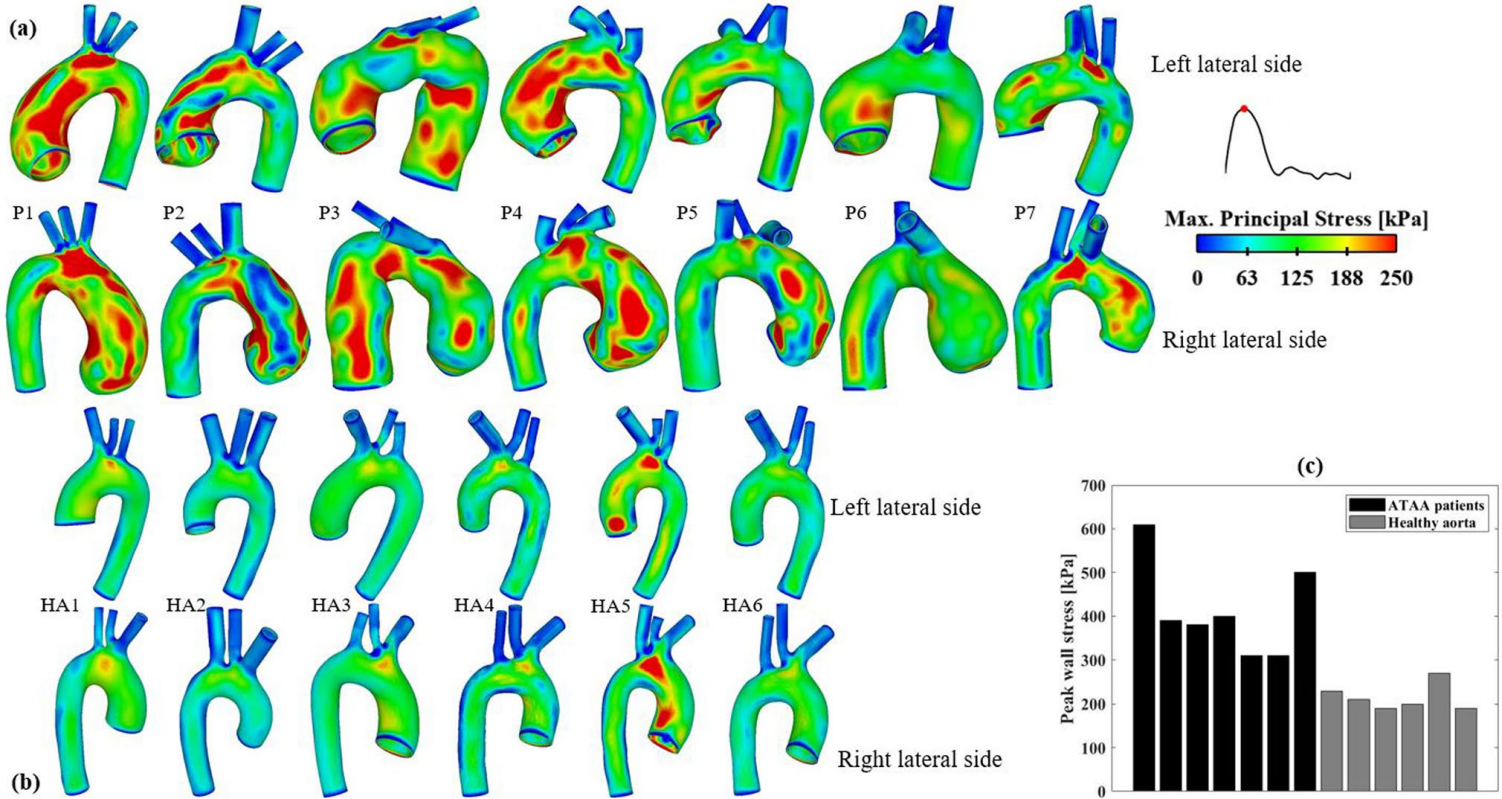
Spatial distributions of maximum principal stress in (**a**) ATAA patients and (**b**) healthy aortas. (**c**) Quantitative comparison of peak wall stress, as represented by the 99th percentile value, between the two groups

Figure 6 displays wall stress distributions at peak systole, highlighting regions with wall stress exceeding 250 kPa in red. In the ATAA models, these high wall stress regions appear mainly in the ascending aorta and aortic arch, except for P3, who also presents with a dilated descending aorta, leading to high wall stress throughout the entire aorta. In contrast, elevated wall stress is observed in only 1 of the 6 healthy aortas. Peak wall stress values, calculated as the 99th percentile of maximum principal stress (MPS), are compared in Fig. 6c. Again, the peak wall stress is much higher in ATAA patients (310 kPa–610 kPa) than in healthy aortas (190 kPa–210 kPa).

### Comparisons between 3D-IVP, Flat-IVP, and Para-IVP

#### Flow patterns and kinetic energy

**Fig. 7:**
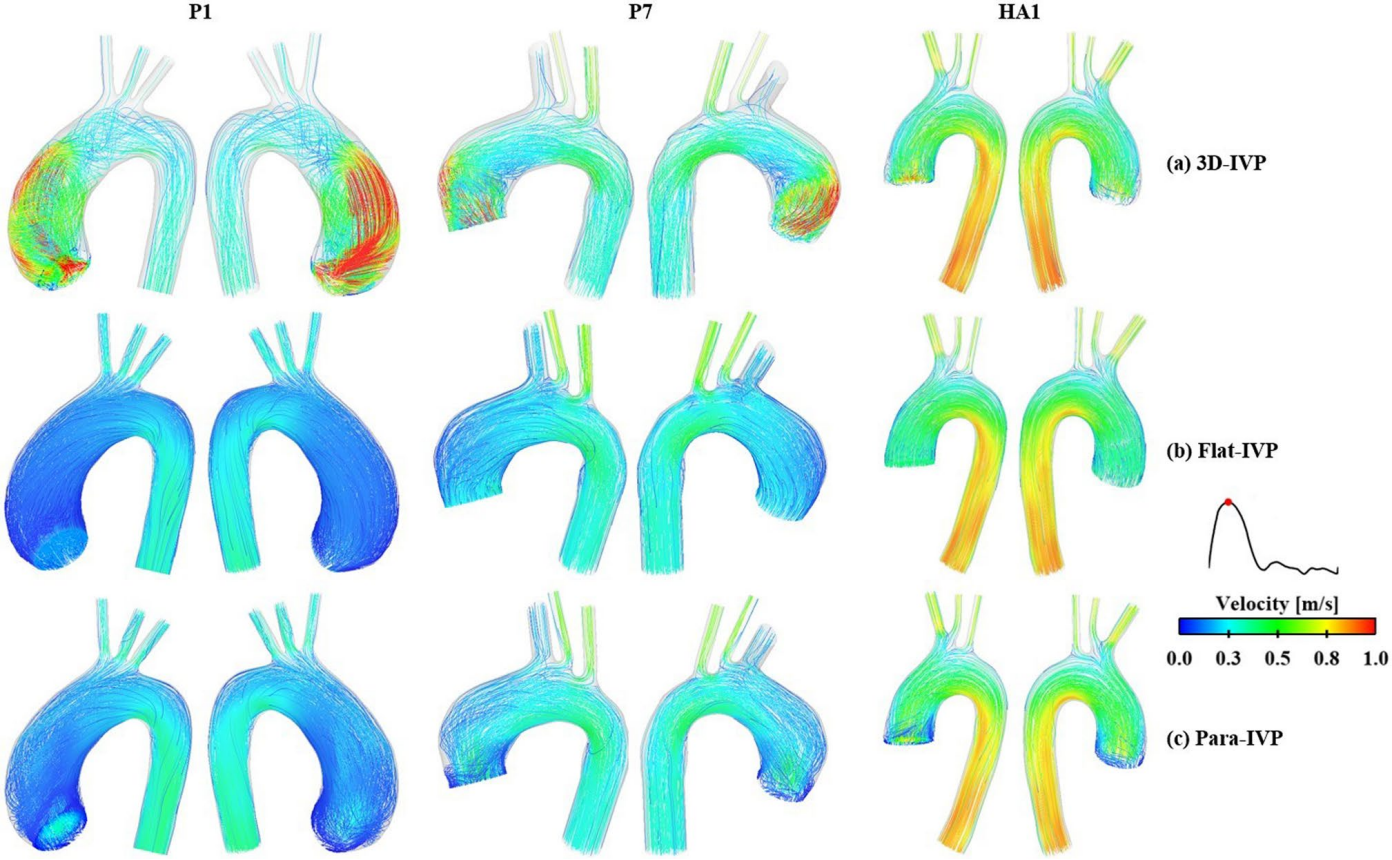
Comparison of instantaneous velocity streamlines at peak systole between the FSI models with (**a**) 3D velocity inlet profiles extracted from 4D flow data, (**b**) flat velocity profiles, and (**c)** parabolic velocity profiles. Three models were selected for additional FSI simulations: P1 and P7 from the group of ATAA patients and HA1 from the group of healthy aortas

Comparisons of velocity streamlines obtained with the patient-specific 3D-IVP and idealized IVPs are shown in Fig. 7. While the peak systolic flow patterns in the healthy aorta are qualitatively similar for simulations with different inlet velocity profiles, dramatic differences are observed in the ATAA models where flow predicted with the 3D-IVP is more eccentric with significantly higher velocities in the proximal ascending aorta than with Flat-IVP and Para-IVP. Both types of idealized IVPs produce more uniformly distributed blood flow patterns throughout the entire aorta. Not surprisingly, the peak KE drops from 5664 Pa to 70 Pa and from 768 Pa to 193 Pa, for P1 and P7, respectively, when using a Flat-IVP, and is reduced to 82 Pa and 184 Pa with the Para-IVP. Similarly, the peak TKE in P1 decreases from 571 Pa to 0.01 Pa with the Flat-IVP and 0.02 Pa with the Para-IVP. In P7, it drops from 73 Pa to 0.01 Pa and 0.08 Pa with the Flat-IVP and Para-IVP, respectively.

#### TAWSS

**Fig. 8:**
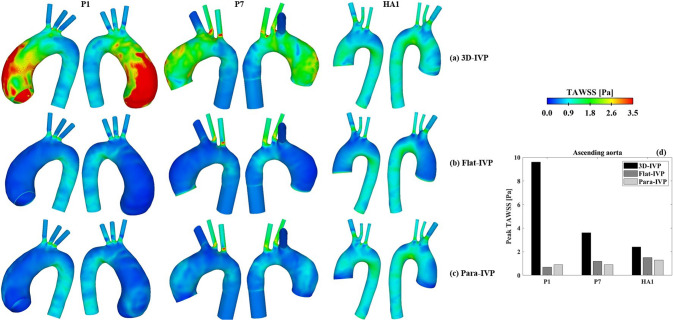
Comparison of time-averaged wall shear stress (TAWSS) distributions between the FSI models with (**a**) 3D velocity inlet profiles extracted from 4D flow data, (**b**) flat velocity profiles, and (**c**) parabolic velocity profiles. (**d**) Quantitative comparison of peak TAWSS in the ascending aorta between the FSI simulations using different inlet velocity profiles

Comparisons of TAWSS obtained with the three types of IVPs are shown in Fig. 8. Overall, adopting idealized IVPs leads to reduced TAWSS in both the ATAA and healthy aorta models, and the reduction is more prominent in the ascending aorta than the descending aorta, and for the ATAA than the healthy aorta. In P1, the peak TAWSS reduces from 9.6 Pa to 3.5 Pa (Flat-IVP) and 3.9 Pa (Para-IVP), respectively, while its location has shifted from the ascending aorta to the ostium of arch vessels. In P7, the peak TAWSS is observed at the ostium of arch vessels across all simulated models, with its magnitude slightly decreasing from 5.1 Pa to 4.4 Pa and 4.2 Pa, with Flat-IVP and Para-IVP, respectively. When examining peak TAWSS specifically in the ascending aorta, it further decreases to 0.7 Pa and 0.9 Pa in P1, for Flat-IVP and Para-IVP, respectively, and from 3.6 Pa to 1.2 Pa (Flat-IVP) and 0.9 Pa (Para-IVP) in P7 (Fig. 8c). Similarly, in the healthy aorta, peak TAWSS in the ascending aorta drops from 2.4 Pa to 1.5 Pa with Flat-IVP and to 1.3 Pa with Para-IVP, whereas the TAWSS distribution obtained with Para-IVP is more comparable to that obtained with the 3D-IVP.

#### Flow asymmetry and dispersion

**Fig. 9:**
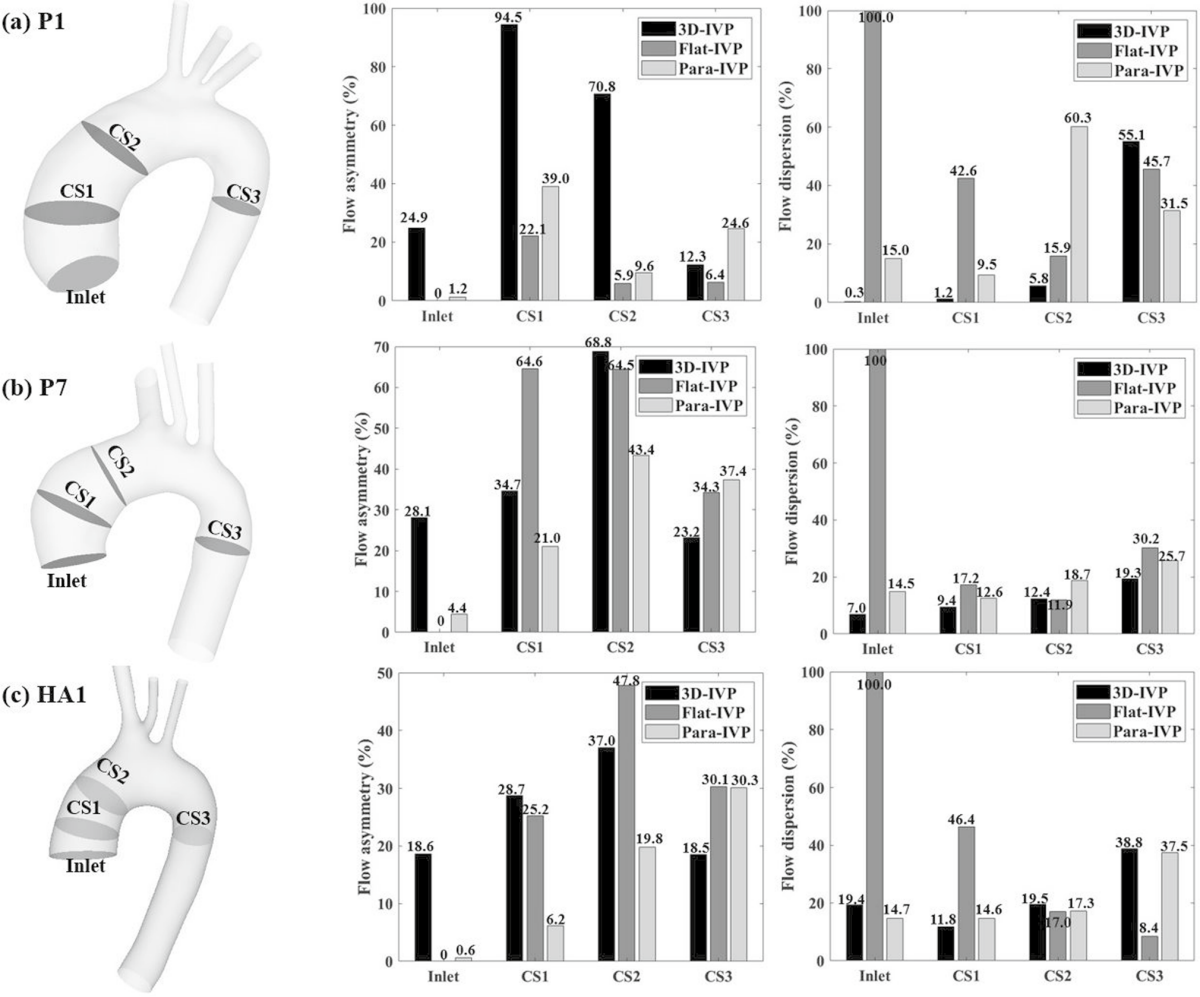
Quantitative comparisons of flow asymmetry and dispersion across different locations in two ATAA models (**a**) P1, (**b**) P7, and (**c**) healthy aorta. Cross-sectional planes 1 (CS1) and 2 (CS2) are located in the ascending aorta, while cross-sectional plane 3 (CS3) is placed in the descending aorta

Figure 9 shows comparisons of ${\text{Flow}}_{{{\text{asymmetry}}}}$ and ${\text{Flow}}_{{{\text{dispersion}}}}$ calculated at the inlet and 3 cross-sectional planes within the two ATAA models and healthy aorta, at the time point of peak systole. In P1, using idealized IVPs leads to substantial reductions in ${\text{Flow}}_{{{\text{asymmetry}}}}$ in the ascending aorta (CS1 and CS2) and corresponding increases in ${\text{Flow}}_{{{\text{dispersion}}}}$ values. In the descending aorta (CS3), the differences are narrower with the Para-IVP resulting in more flow eccentricity than the 3D-IVP and Flat-IVP. In P7, using Flat-IVP results in either increased (CS1) or comparable (CS2) flow eccentricity in the ascending aorta, while adopting Para-IVP leads to moderate reductions in ${\text{Flow}}_{{{\text{asymmetry}}}}$ throughout the ascending aorta. In the descending aorta (CS3), using idealized IVPs increases ${\text{Flow}}_{{{\text{asymmetry}}}}$ with values obtained from the two idealized IVPs being comparable. Regarding ${\text{Flow}}_{{{\text{dispersion}}}}$, the discrepancies are smaller with both idealized IVPs leading to a broader distribution of peak velocities. In the healthy aorta, comparable ${\text{Flow}}_{{{\text{dispersion}}}}$ values are observed between the 3D-IVP and Para-IVP throughout the aorta, but ${\text{Flow}}_{{{\text{asymmetry}}}}$ values differ considerably, especially in the ascending aorta. In the descending aorta, ${\text{Flow}}_{{{\text{asymmetry}}}}$ values obtained with the two idealized IVPs are comparable.

#### Wall stress

**Fig. 10:**
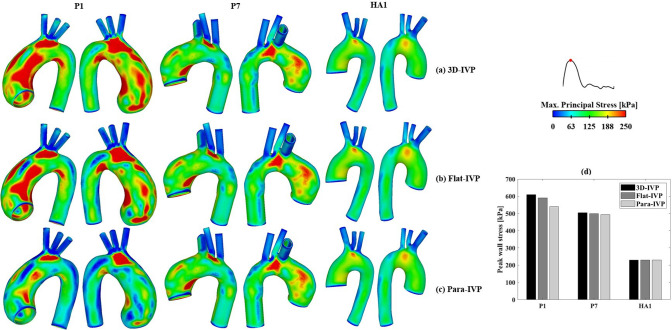
Comparison of spatial distributions of maximum principal stress (MPS) between the FSI models with (**a**) 3D velocity inlet profiles extracted from 4D flow data, (**b**) flat velocity profiles, and (**c**) parabolic velocity profiles. (**d**) Quantitative comparison of peak wall stress, as presented by the 99th percentile of MPS, between the FSI simulations using different inlet velocity profiles

Figure 10 presents a qualitative comparison of spatial distributions of MPS. In both the healthy aorta and P7, the MPS distributions are comparable between the results obtained with different IVPs, whereas notable differences are observed in P1 with severe aortic valve stenosis. In P1, using idealized IVPs results in substantial reductions in regions with MPS > 250 kPa, particularly with the Para-IVP. These reductions are primarily localized along the inner and outer curvatures of the aneurysmal aorta. Quantitative comparison of peak wall stress (as represented by the 99th percentile of MPS, Fig. 10d) illustrates a similar reduction trend in both ATAA cases when using idealized IVPs, with peak wall stress values decreasing from 610 kPa (3D-IVP) to 590 kPa (Flat-IVP) and 540 kPa (Para-IVP) in P1, and from 504 kPa (3D-IVP) to 499 kPa (Flat-IVP) and 494 kPa (Para-IVP) in P7. In contrast, peak wall stress remains consistent across different IVPs in the healthy aorta.

## Discussion

This study demonstrates significant differences in hemodynamic and biomechanical metrics between ATAA models and healthy aortas using two-way FSI simulations under patient-specific flow conditions. Our findings indicate that ATAA patients are characterized by more disturbed blood flow, significantly elevated TKE, TAWSS, and peak wall stress compared to healthy controls. Importantly, patient-specific 3D-IVP extracted from 4D flow MRI proved critical for accurately predicting these metrics, particularly in ATAA cases. These results reinforce the value of FSI modeling combined with 4D flow MRI in personalized risk stratification.

From a biomechanical perspective, rupture or dissection occurs when the local stresses exceed the mechanical strength of the aortic wall. Several studies have suggested that peak wall stress could serve as a potential predictor for the rupture risk of ATAAs (Campobasso et al. 2018; García-Herrera et al., 2017; Trabelsi et al. 2015; Wisneski et al. 2014). In the present study, the peak wall stress in ATAA models varied between 310 kPa and 610 kPa, which is well below the experimentally measured rupture stresses of 760 kPa–2330 kPa (Trabelsi et al. 2015). However, this does not necessarily mean that these ATAA cases would be at low risk of rupture. The study of Trabelsi et al., (2015) also reported that the peak wall stress values predicted by FEA under normal pressure conditions were within 27%–75% of the corresponding rupture stresses, suggesting that rupture or dissection might occur at locations other than those with the peak wall stress, particular in regions where the tissue is significantly weaker. This has been demonstrated by a previous study, which revealed substantial regional variations in mechanical properties and strength of ATAA tissues (Salmasi et al. 2022).

Acting directly on the endothelium, WSS can induce altered adaptive processes and microstructural changes that contribute to the weakening of the aortic wall (Redheuil et al. 2011). Several studies have combined 4D flow MRI with CFD to investigate the relationship between WSS and biomechanical properties of the aortic wall in patients with ATAAs (Condemi et al. 2017, 2019; Gülan et al. 2018; Jayendiran et al. 2020; McClarty et al. 2022; Mousavi et al. 2021; Ramaekers et al. 2024; Salmasi et al. 2021). High WSS was found not only to indicate an increased risk of aortic dissection by reducing longitudinal dissection energy (Salmasi et al. 2021) and delamination strength (McClarty et al. 2022), but also to compromise aortic wall integrity by lowering elastin levels and smooth muscle cell counts (Salmasi et al. 2021). Condemi et al. (2017, 2019) conducted two separate studies. Although a direct correlation between high TAWSS and reduced tissue strength was not identified in their early study (Condemi et al. 2017), their later study revealed that high TAWSS was linked to increased rupture stretch, indicating a higher risk of rupture (Condemi et al. 2019). In the present study, elevated TAWSS was observed in regions of the aneurysm where blood flow impinges on the aortic wall (Fig. 5), suggesting that these regions may be prone to rupture. TAWSS distributions in the healthy aortas were more uniform, with elevated TAWSS mainly observed in the arch vessels and along the inner curvature of the distal arch. Specifically for the ascending aorta, the peak TAWSS ranged between 2.0 Pa and 3.2 Pa in the healthy aortas (2.8 ± 0.7 Pa), which is within the reported range of 1–7 Pa (Malek et al. 1999). The maximum TAWSS values (8.6 ± 6.5 Pa) were significantly higher in the ATAA cohort.

Barker et al. (2018) hypothesized that co-localization of abnormal WSS and high wall stress on the aneurysmal wall might help predict rupture. However, no appropriate thresholds for defining abnormal high WSS and wall stress in the aorta can be found in the literature, especially when both metrics are considered simultaneously. In the present study, by examining Figs. 5 and 6 together, regions simultaneously exposed to elevated TAWSS (> 3.5 Pa) and elevated MPS (> 250 kPa) were identified in 6 of the 7 ATAA models. It was also interesting to note that the predicted peak wall stress in the aneurysmal aorta could be influenced by the choice of IVP. In both ATAA models, the peak wall stress in the ascending aorta was lower with idealized IVPs, decreasing by 3.3% and 11.5% in P1 with Flat-IVP and Para-IVP, respectively, and by 1.0% and 2.0% in P7, respectively, compared to 3D-IVP. The observed reduction in peak wall stress was mainly caused by the reduced pressure load in the ascending aorta of the ATAA models, where spatial-mean pressure at peak systole was 122.1 mmHg, 111.2 mmHg, and 96.3 mmHg, for 3D-IVP, Flat-IVP, and Para-IVP, respectively, in P1, and was 111.1 mmHg. 110.7 mmHg, and 109.2 mmHg, respectively, in P7. Additionally, the more pronounced impact of using idealized IVPs in P1 than in P7 could be attributed to their different types and severity of aortic valve diseases: in P1 with severe aortic stenosis, the maximum jet velocity reached 4.2 m/s but decreased to 0.16 m/s with Flat-IVP and 0.32 m/s with Para-IVP, whereas in P7 with mild regurgitation, the much lower peak velocity of 1.5 m/s was reduced to 0.25 m/s and 0.49 m/s with Flat-IVP and Para-IVP, respectively. For the healthy aorta, the impact of using idealized IVPs was less pronounced, with the peak wall stress remaining the same across all the simulated models.

Moreover, our results highlighted the importance of employing patient-specific 3D-IVP at the inlet for accurately capturing the location of high TAWSS. Albeit comparable results were obtained with all three types of IVPs for the healthy case, significant differences were noted in both ATAA cases, where the high-velocity gradient resulting from the fast and eccentric inflow jet was not captured by any of the idealized IVPs. This could be explained by the presence of significant secondary flows in the ATAA patients, which were neglected by both the Flat- or Para-IVPs. At peak systole, using the normal velocity component alone underestimated the mean total velocity derived from 4D flow MRI by 77.1% in P1, 52.1% in P7, and 18.8% in the healthy aorta. Clearly, pronounced secondary flows were present in the ATAA models, even in P7 with mild aortic regurgitation. This is consistent with the findings by Pirola et al. (2018). To better understand the underlying mechanisms, we also evaluated the flow eccentricity (${\text{Flow}}_{{{\text{asymmetry}}}}$) and dispersion (${\text{Flow}}_{{{\text{dispersion}}}}$) levels at the time point of peak systole at several cross-sectional planes in both the ascending and descending aorta (Fig. 9). Our results suggest that compared to hemodynamic indices in the descending aorta, those in the ascending aorta are more sensitive to the choice of IVP, supporting the findings by Youssefi et al. (2018).

Specifically for the ATAA cases, the highly skewed high-speed flow jet in the ascending aorta could only be captured by 3D-IVP. These findings emphasize the necessity of applying patient-specific 3D-IVP for ATAA patients to further investigate the predictability of co-localization of abnormal WSS and high wall stress on the aneurysmal wall in future studies. In P1, resulting from the significantly reduced skewness in blood flow, the spatial-mean TAWSS across the aneurysmal wall was only 0.3 Pa and 0.4 Pa with a Flat-IVP and Para-IVP, respectively, which is an order of magnitude lower than the value predicted by 3D-IVP. Although less pronounced in P7, the spatial-mean TAWSS was reduced from 1.8 Pa to 0.4 Pa with Flat-IVP and 0.6 Pa with Para-IVP. Therefore, in cases where patient-specific 4D flow MR images are unavailable, the synthetic 3D-IVP reported in the study by Saitta et al. (2023) should be adopted as an alternative to idealized IVPs.

In a previous CFD study of one ATAA and one healthy aorta, TKE of the aneurysmal aorta was found to be around 2.5 times higher than that of the healthy aorta, suggesting that monitoring elevated TKE in the follow-up of ATAA patients may help predict aneurysm progression (Gülan et al. 2018). In our study, TKE at peak systole was calculated and plotted across the entire aortic lumen. The peak TKE averaged among 7 ATAA patients (155 Pa) was over 250 times higher than that of 6 healthy aortas (0.6 Pa). However, it should be noted that TKE was spatially and temporally averaged over two cross-sectional planes in Gülen et al.’s study, whereas peak systolic TKE was compared in our study. Moreover, our study considered the impact of wall deformation, which may significantly impact regions with low WSS (Zhu et al. 2022). The TKE predicted in the current study for ATAA patients agrees better with another study that applied large-eddy simulation to a patient with aortic valve disease and a dilated ascending aorta (Manchester et al. 2021). High levels of TKE were observed in regions between highly skewed jet and the surrounding low-velocity blood, areas that could not be captured by either of the idealized IVPs. Consequently, in both ATAA cases, the maximum TKE was substantially reduced to below 0.1 Pa when using either a Flat-IVP or Para-IVP, which is even lower than that of healthy aortas.

### Limitations

The FSI simulations presented in this study had several assumptions. First, the aortic wall was assumed to have a constant and uniform thickness, despite substantial regional variations in aortic thickness having been demonstrated (Salmasi et al. 2022). Previous FEA studies showed that incorporating local wall thickness could significantly increase peak wall stress magnitudes and alters their regional distributions (Shang et al. 2013). However, due to insufficient detail in CT images for wall thickness measurement, assuming a uniform wall thickness remains common practice (Campobasso et al. 2018; García-Herrera et al. 2017; Trabelsi et al. 2015; Wisneski et al. 2014). Second, it is well established that the mechanical behavior of aortic wall is anisotropic and nonlinear, and an anisotropic constitutive model developed by Holzapfel et al. (2000) has been applied in previous FE studies (Wang et al. 2021; Xuan et al. 2018). However, employing an anisotropic wall model would further increase the computational costs of FSI simulations. Therefore, an isotropic hyperelastic material was adopted in the present study which involved 19 FSI simulations. Third, different imaging modalities were used to reconstruct geometric models of the ATAA and healthy aorta as CT scans of healthy aortas were not available. Despite the relatively low spatial resolution of 4D flow MR images, they were sufficient to capture all the important geometric features of a healthy aorta. However, the reconstructed aortic diameter would be less accurate than that from the CT scans, which would affect the predicted wall shear stress values. This is unlikely to change the main findings of this study, as the difference in aortic diameter between the ATAA models and healthy controls is much larger than the uncertainties in reconstructing the healthy aorta geometry. Additionally, extraction of the IVP from 4D flow data can incur small errors during diastole where velocities are picked up outside of the aortic lumen due to the fixed area of the inlet plane. However, this has minimal effect on results compared to the large volume of systolic flow. Fourth, only two ATAA cases and one healthy aorta were selected to assess the impact of using idealized IVPs. However, the two chosen ATAA patients are representative, and we would anticipate similar findings if the same FSI simulations were performed across the other patients, as using idealized IVPs would consistently neglect secondary flows, which are essential for accurately capturing hemodynamics in patients with aortic valve pathologies. Finally, the aortic root motion was neglected. Jin et al. (2003) reported that incorporating both radial expansion–contraction and translational motion of the aorta at the inlet in their CFD model produced the results that best matched the in vivo MR data. On the other hand, Singh et al. (2016) found that including aortic root downward motion in their FE model significantly increased the longitudinal stress in the ascending aorta. Therefore, the influence of aortic root motion should be considered in future computational studies.

## Conclusion

Fully coupled two-way FSI simulations incorporating hyperelastic aortic wall properties, prestress, and patient-specific 3D-IVP were performed on 7 ATAA patients, with results compared to those from 6 healthy aortas. The blood flow was significantly more disturbed in the ATAA models, leading to much greater TKE and TAWSS. In addition, peak wall stress was markedly higher in ATAA walls compared to the healthy aortas. The substantial differences in simulation results between the two groups indicate that regions with altered wall mechanics and abnormal hemodynamics could be captured simultaneously through FSI simulations. Notably, patient-specific 3D-IVP proved crucial in capturing regions of high TKE, elevated TAWSS, and high wall stress that were either undetected or underestimated when using a Flat-IVP or Para-IVP. Future studies with large patient cohorts or longitudinal data will be necessary to further validate the role of FSI modeling combined with 4D flow MRI in risk stratification for ATAA patients.

## Supplementary Information

Below is the link to the electronic supplementary material.

Supplementary file1 (DOCX 26 KB)

## Data Availability

All the data support findings of this study are included in the article/Supplementary Material, further inquiries can be directed to the corresponding author.
