# Supplementary material for: A combined 4D flow MR imaging and fluid–structure interaction analysis of ascending thoracic aortic aneurysms
Source: Biomech Model Mechanobiol. 2025 Mar 11;24(3):829–44. doi: 10.1007/s10237-025-01939-6 (PMC12162810; doi:10.1007/s10237-025-01939-6)
Supplement: Supplementary file 1 — Supplementary file1 (DOCX 26 KB) [file 10237_2025_1939_MOESM1_ESM.docx]

**A combined 4D flow MR imaging and fluid-structure interaction analysis of ascending thoracic aortic aneurysms**

Yu Zhu^a^, [Chlöe Armour](https://journals.sagepub.com/doi/10.1177/15266028221111295#con1)**^a,b^**, Binghuan Li**^a^,** Selene Pirola**^c^**, Yousuf Salmasi^d^, Thanos Athanasiou^d^, Declan P. O’Regan**^e^**, Xiao Yun Xu**^a*^**

a. Department of Chemical Engineering, Imperial College London, UK

b. National Heart and Lung Institute, Imperial College London, UK

c. Department of Biomechanical Engineering, Delft University of Technology, Netherlands

d. Department of Surgery and Cancer, Imperial College London, UK

e. MRC London Institute of Medical Sciences, Imperial College London, UK

*** Correspondence:**Professor Xiao Yun Xu [yun.xu@imperial.ac.uk](mailto:yun.xu@imperial.ac.uk)

# **Statements and Declarations**

**Competing Interests:** All authors have nothing to disclose.

# **S1. Mesh sensitivity tests**

Mesh sensitivity tests were performed for fluid and structural domains, separately, to ensure that a mesh independent solution has been achieved.

In terms of fluid domain, transitional flow simulations were conducted using different meshes but with identical boundary conditions and simulation settings to allow for direct comparison of results. It should be noted that a flat velocity profile was used as the inlet boundary condition to reduce computational time. The peak systolic velocity and wall shear stress (WSS) were spatially averaged across two selected planes, as well as the entire domain for one ATAA model and one healthy aorta, with their maximum values being compared among different meshes. For the ATAA model, the two cross-sectional planes were located at the ascending aorta, whereas for the healthy aorta, one plane was placed in the ascending aorta and the other in the aortic arch.

As proposed by Craven et al., the mesh sensitivity study can be quantitatively conducted by evaluating the grid convergence index, GCI, on three sets of mesh with the increasing number of elements on each set (Craven et al., 2009). Starting by defining the target number of elements on each mesh with a grid refinement ratio:

$$r= {(\frac{N_{3}}{N_{2}})}^{1/D}= {(\frac{N_{2}}{N_{1}})}^{1/D}$$

where $N_{1,2,3}$ denotes the number of elements in mesh M1 (coarse), M2 (medium), and M3 (fine), and *D* = 3 is the dimension of the flow field.

The order of convergence was calculated based on the evaluated flow quantities:

$$p=\ln(\frac{\left| f_{1}- f_{2} \right|}{\left| f_{2}- f_{3} \right|})/\ln(r)$$

where $f_{1, 2,3}$ is the analysed flow quantities, namely, the peak spatial-mean velocity and WSS.

Then, the error estimator of each quantity between every two sets of mesh can be defined as:

$$\varepsilon_{1,2}=\frac{\left( \frac{\left| f_{1}- f_{2} \right|}{f_{2}} \right)}{r^{p}-1}, \varepsilon_{2,3}= \frac{(\frac{\left| f_{2}- f_{3} \right|}{f_{3}})}{r^{p}-1}$$

Finally, the GCI can be calculated as:

$${GCI}_{1,2}= F_{S}\left| \varepsilon_{1,2} \right|, {GCI}_{2,3}= F_{S}\left| \varepsilon_{2,3} \right|$$

where $F_{S}$ is the ‘factor of safety’ equal to 1.25, when comparing for more than 2 meshes (Craven et al., 2009).

The chosen mesh for each model had an error ($\varepsilon$) of < 3.5% for each hemodynamic parameter between the selected mesh and a more refined mesh, as well as a grid convergence index (GCI) of < 5.5%, in line with previous studies (Armour et al., 2021; Craven et al., 2009). The results of mesh sensitivity tests for the fluid domains of both patients are summarized in Table S1. It is clear that M2 containing approximately 2.64 and 1.16 million elements for ATAA model and healthy aorta, respectively, should be adopted for final simulations.

Table S1. Mesh sensitivity tests by GCI showing comparison of velocity and wall shear stress (WSS) between different meshes for the fluid domain.

| ATAA model | | | | |
| --- | --- | --- | --- | --- |
|  | Number of elements | P1 (Spatial mean) | P2 (Spatial mean) | Entire domain (Spatial mean) |
|  |  | Max. velocity at peak systole (m/s) | | |
| M1 | 890104 | 0.514 | 0.563 | 0.376 |
| M2 | 2641910 | 0.522 | 0.580 | 0.375 |
| M3 | 5207829 | 0.516 | 0.575 | 0.376 |
| $\varepsilon_{1,2}$ (%) | | 3.667 | 1.213 | 1.490 |
| $\varepsilon_{2,3}$ (%) | | 2.589 | 0.353 | 1.399 |
| ${GCI}_{1,2}$ (%) | | 4.584 | 1.516 | 1.863 |
| ${GCI}_{2,3}$ (%) | | 3.236 | 0.441 | 1.745 |
|  | | Max. WSS at peak systole (Pa) | | |
| M1 | 890104 | 6.242 | 5.330 | 2.642 |
| M2 | 2641910 | 6.491 | 5.542 | 2.747 |
| M3 | 5207829 | 6.541 | 5.607 | 2.742 |
| $\varepsilon_{1,2}$ (%) | | 0.945 | 1.671 | 0.173 |
| $\varepsilon_{2,3}$ (%) | | 0.185 | 0.501 | 0.008 |
| ${GCI}_{1,2}$ (%) | | 1.181 | 2.089 | 0.216 |
| ${GCI}_{2,3}$ (%) | | 0.231 | 0.626 | 0.009 |
| Control case | | | | |
|  | Number of elements | P1 (Spatial mean) | P2 (Spatial mean) | Entire domain (Spatial mean) |
|  |  | Max. velocity at peak systole (m/s) | | |
| M1 | 328820 | 0.609 | 0.469 | 0.538 |
| M2 | 115865 | 0.619 | 0.468 | 0.539 |
| M3 | 2566044 | 0.625 | 0.468 | 0.540 |
| $\varepsilon_{1,2}$ (%) | | 0.533 | 0.024 | -0.624 |
| $\varepsilon_{2,3}$ (%) | | 0.283 | 0.002 | -0.790 |
| ${GCI}_{1,2}$ (%) | | 0.666 | 0.030 | 0.780 |
| ${GCI}_{2,3}$ (%) | | 0.354 | 0.003 | 0.987 |
|  | | Max. WSS at peak systole (Pa) | | |
| M1 | 328820 | 4.137 | 3.846 | 3.316 |
| M2 | 115865 | 4.374 | 3.797 | 3.289 |
| M3 | 2566044 | 4.462 | 3.770 | 3.278 |
| $\varepsilon_{1,2}$ (%) | | 3.169 | 1.716 | 0.538 |
| $\varepsilon_{2,3}$ (%) | | 1.145 | 0.989 | 0.213 |
| ${GCI}_{1,2}$ (%) | | 3.961 | 2.146 | 0.672 |
| ${GCI}_{2,3}$ (%) | | 1.431 | 1.236 | 0.266 |

Regarding the structural domain, static structural analyses were performed for different meshes to compare the results of peak maximum and middle principal stress across the entire aortic wall. Solutions were considered mesh independent when the GCI was less than 5.5% between two successively refined meshes. The results of mesh sensitivity tests for the structural domains of both models are summarized in Table S2. The results show that M2 of both models should be chosen for final simulations.

Table S2. Mesh sensitivity tests by GCI showing comparison of the peak maximum and middle principal stress between different meshes for the structural domain.

| ATAA model | | |
| --- | --- | --- |
|  | Number of elements | Peak max. principal stress (MPa) |
| M1 | 305701 | 0.863 |
| M2 | 1181934 | 0.892 |
| M3 | 3093805 | 0.906 |
| $\varepsilon_{1,2}$ (%) | | 3.110 |
| $\varepsilon_{2,3}$ (%) | | 1.497 |
| ${GCI}_{1,2}$ (%) | | 3.887 |
| ${GCI}_{2,3}$ (%) | | 1.870 |
|  | | Peak mid. Principal stress (kPa) |
| M1 | 305701 | 0.147 |
| M2 | 1181934 | 0.190 |
| M3 | 3093805 | 0.203 |
| $\varepsilon_{1,2}$ (%) | | 9.810 |
| $\varepsilon_{2,3}$ (%) | | 2.774 |
| ${GCI}_{1,2}$ (%) | | 12.262 |
| ${GCI}_{2,3}$ (%) | | 3.468 |
| Control case | | |
|  | Number of elements | Peak max. principal stress (kPa) |
| M1 | 243164 | 0.330 |
| M2 | 792453 | 0.337 |
| M3 | 2444247 | 0.342 |
| $\varepsilon_{1,2}$ (%) | | 5.673 |
| $\varepsilon_{2,3}$ (%) | | 4.078 |
| ${GCI}_{1,2}$ (%) | | 7.091 |
| ${GCI}_{2,3}$ (%) | | 5.097 |
|  | | Peak mid. principal stress (kPa) |
| M1 | 243164 | 0.221 |
| M2 | 792453 | 0.226 |
| M3 | 2444247 | 0.226 |
| $\varepsilon_{1,2}$ (%) | | 0.454 |
| $\varepsilon_{2,3}$ (%) | | 0.075 |
| ${GCI}_{1,2}$ (%) | | 0.568 |
| ${GCI}_{2,3}$ (%) | | 0.093 |

# **References**

Armour, C.H., Guo, B., Pirola, S., Saitta, S., Liu, Y., Dong, Z., Xu, X.Y., 2021. The influence of inlet velocity profile on predicted flow in type B aortic dissection. Biomech. Model. Mechanobiol, 20, 481–490. https://doi.org/10.1007/s10237-020-01395-4

Craven, B.A., Paterson, E.G., Settles, G.S., Lawson, M.J., 2009. Development and verification of a high-fidelity computational fluid dynamics model of canine nasal airflow. J. Biomech. Eng. 131, 091002. <https://doi.org/10.1115/1.3148202>
